# A Public Domain Dataset for Real-Life Human Activity Recognition Using Smartphone Sensors

**DOI:** 10.3390/s20082200

**Published:** 2020-04-13

**Authors:** Daniel Garcia-Gonzalez, Daniel Rivero, Enrique Fernandez-Blanco, Miguel R. Luaces

**Affiliations:** Department of Computer Science and Information Technologies, University of A Coruna, 15071 A Coruna, Spain; daniel.rivero@udc.es (D.R.); enrique.fernandez@udc.es (E.F.-B.); miguel.luaces@udc.es (M.R.L.)

**Keywords:** HAR, human activity recognition, sensors, smartphones, dataset, SVM

## Abstract

In recent years, human activity recognition has become a hot topic inside the scientific community. The reason to be under the spotlight is its direct application in multiple domains, like healthcare or fitness. Additionally, the current worldwide use of smartphones makes it particularly easy to get this kind of data from people in a non-intrusive and cheaper way, without the need for other wearables. In this paper, we introduce our orientation-independent, placement-independent and subject-independent human activity recognition dataset. The information in this dataset is the measurements from the accelerometer, gyroscope, magnetometer, and GPS of the smartphone. Additionally, each measure is associated with one of the four possible registered activities: inactive, active, walking and driving. This work also proposes asupport vector machine (SVM) model to perform some preliminary experiments on the dataset. Considering that this dataset was taken from smartphones in their actual use, unlike other datasets, the development of a good model on such data is an open problem and a challenge for researchers. By doing so, we would be able to close the gap between the model and a real-life application.

## 1. Introduction

Giving birth to the knowledge area called human activity recognition (HAR), the accurate identification of different human activities has become a hot research topic. This area tries to identify the action performed by a subject based on the data records from a set of sensors. The recording of these sensors is carried out while the subject performs a series of well-defined movements, such as nodding, raising the hand, walking, running or driving. In this sense, wearable devices, such as activity bracelets or smartphones, have become of great use as sources of this sort of data. This kind of devices, especially the latter ones, provide a broad set of sensors in a convenient size which can be used relatively easy with high-grade performance and accuracy. The researchers use the information about people’s behaviors gathered by these sensors to support the demands from domains like healthcare, fitness or home automation [1]. The result from the intersection between the widespread sensing all over the world, due to the smartphones and the models developed from that continuous recording, is a research area that has attracted increasing attention in recent years [2].

The main challenges to be tackled are two: first, managing the vast number of information that the devices can produce, as well as their temporal dependency, and, second, the lack of knowledge about how to relate this data to the defined movements. Some methods have achieved remarkable results in extracting information from these sensors readings [3,4]. However, it is relevant to note that in such studies, the devices have been modified to be carried in a particular way, attached to different body parts, such as waist or wrist. Therefore, the success of those models can be biased using data collected in such a controlled environment, with specific device orientations and a few activities. Regarding these orientations, this is far from the ideal scenario, as every person may use these devices, especially their smartphones, in many different ways. For the same individual, different clothes may vary the orientation and placement of the device. In the same way, for different individuals, their body shape, as well as their behavior, can make an enormous difference too. In this way, the artificial intelligence (AI) models proposed to date are highly dependent on orientation and placement. For that reason, they cannot be generalized to every kind of user, so there has not been a real transition to real-life, yet. Presently, personalization of AI models in HAR for large numbers of people is still an active research topic [5,6], despite being actively researched for nearly a decade [7,8].

To address the aforementioned issues, this work presents a more realistic dataset which is independent of the device orientation and placement, while it also keeps the independence of the user. Those are the main differences according to data with other works developed so far. Additionally, with the implementation of a simple support vector machine (SVM) model, we present a first model as proof of concept to detect the main activities in the more realistic dataset. In this way, we are laying the foundations for the transition of this type of system into real life.

Therefore, the main contributions of this paper can be summed up as follows:

- Provide and make publicly available a new HAR dataset closer to a realistic scenario (see the files in Supplementary Materials). This dataset is independent of the device orientation and placement, while it is also individual independent.
- The new dataset adds additional signals not very explored until today like the GPS and magnetometer sensor measurements.
- A first reference model is provided for this dataset, after applying a specific sliding window length and overlap.
- A study of the best architecture for longer-themed activities, such as those suggested in our work.

The organization of the rest of the paper is as follows. Section 2 shows some related works on HAR, as well as other datasets used in this field. Section 3 gives a thorough explanation of the dataset arrangement, as well as the data collection process. Section 4 presents and discuss the experimental results obtained on the SVM model we propose, using our custom dataset; while finally, Section 5 contains the conclusions and future work lines.

## 2. Related Work

Inside HAR knowledge area, other datasets have been previously published. The first one worth to mention, because its widespread use in different works and comparisons, is UCI (University of California, Irvine) HAR dataset. Proposed in [9], the dataset contains data gathered while carrying a waist-mounted smartphone with embedded inertial sensors. The time signals, in this case, were sampled in sliding windows of 2.56 s and 50% overlap between them, as the activities researched are done in short intervals of time: standing, sitting, laying down, walking, walking downstairs and walking upstairs. In this work, they also created an SVM model to be exploited. With a total of 561 features extracted, they got particularly good results, with accuracies, precisions and recalls higher than 90%. However, it is a dataset taken in a laboratory environment, with a particular position and orientation. For that reason, in a realistic environment in which users could use their smartphones in their way, the results obtained would not be trustable.

Apart from the UCI HAR dataset, there is the WISDM (Wireless Sensor Data Mining) one [10], which is also widely used. In this case, the sliding windows chosen were of 10 s, with apparently no overlap applied. They mention that they also worked with 20 s, but the results were much better with the first case. Here, the activities researched were: walking, jogging, ascending stairs, descending stairs, sitting and standing. In their work, they used some WEKA (Waikato Environment for Knowledge Analysis) algorithms like J48 or Logistic Regression to perform some predictions over their data, with quite good outcomes. Nonetheless, it has the same problem as the previous case, so its results could not be taken to a real-life environment either.

To highlight these differences, we show in Table 1 a qualitative comparison between these two datasets and the one we propose in this paper.

In the literature, many works tested and validated these datasets. For example, in [11], they made a comparison between Convolutional Neural Networks (CNN), Random Forest, Principal Component Analysis (PCA) and K-Nearest Neighbors (KNN) based algorithms. They concluded that CNN outperforms the rest of the ones they tested, apart from seeing that larger sliding windows did not necessarily improve their behavior. Also, they proposed some CNN architectures, making a comparison between different combinations of hyperparameters and the performance they achieved. Similarly, more recently, [12] also proposed a CNN model to address the HAR problematic, with apparently slightly better results. On the other hand, [13] submitted a combination between feature selection techniques and a deep learning method, concretely a Deep Belief Network (DBN), with some good results, higher than the ones achieved with SVM-based models, which showed to be one of the best algorithms to use in HAR problematics. By contrast, in [14,15] they made comparisons between different feature selections for different widely used machine learning (ML) algorithms in the literature. Results showed that frequency-based features are more feasible, at least for algorithms like SVM or CNN, as they throw the best results.

Furthermore, many other works built their dataset to carry out their research. One of the most interesting ones is [16]. In their work, they propose an online SVM model approach for nine different smartphone orientations. Regarding the data collection, they took it while carrying the mobile in a backpack. On the opposite hand, they also made a comparison between their custom approach and some other generic classifiers, such as KNN, decision trees, and Naive Bayes. These methods, alongside some other techniques like SVM, CNN, Random Forest, and Gradient Boosting, showed to be valid for HAR with a reasonable size of data. In the end, their approach outperformed the rest of the classifiers, but they addressed that the future of HAR would be in deep learning methods, as they seem to get better results in practice. More recent works, like [17,18] show similar results. In these cases, more sensors apart from accelerometer and gyroscope were used, like GPS or magnetometer, showing their potentiality in more long-themed activities like walking or jogging.

Following the same line, other works made their datasets but applying purely Deep Learning methods. In [19], the results show that these methods might be the future for HAR, as their results are very hopeful, at least in the non-stationary activities such as walking or running, as SVM still reigns in short-timed activities such as standing or laying down. More recently, works implementing LSTM (long short-term memory) models are arising. The principal advantage of these implementations is that they take into account past information and, at being a deep learning-based technique, they do not need a prior feature extraction to perform the training. The downside is that they need big datasets to get reliable classification results, as well as more time to be trained and suitable stop criteria to avoid overfitting (and underfitting). For example, in [20,21] we can see this kind of models and with particularly good results. In fact, in [20] they implemented a modification of LSTMs which are called Bi-LSTMs (bidirectional LSTMs). What makes this modification special is that these models can also learn from the future, throwing accuracies of around 95%.

However, as we already addressed in the introduction, all these works depend on a particular device orientation to get these successful results. In [22], the problem of different device orientations, as well as different smartphone models, was addressed. In this case, they got good results by transforming the phone’s coordinate system to the earth coordinate system. Moreover, their results did not show remarkable decreases in accuracy when carrying different smartphone models, but only when the orientation changed. Even so, it does not address the problem that arises when the smartphone is put in different places and not only in the pocket (for example, a bag).

As can be seen, there are problems of lack of realism and applicability in real life of the systems and datasets developed so far in HAR. While the results of many of the models developed in this field are quite promising, their real-life application would probably not be as successful. Therefore, in our work, we are determined to know these problems with the formation of our own more realistic dataset. With a simple SVM model, we could see the performance differences concerning other works and overcome them in future developments, if there are many.

## 3. Materials and Methods

This part contains a step-by-step description of our work, divided into the following sections. First, Section 3.1 presents the procedure carried out to collect the data. Then, in Section 3.2, we describe how the data was prepared to use once the data collection was over, as well as the features extracted from them. Finally, Section 3.3 offers a summary of the classification algorithm applied.

The dataset and all the resources used in this paper are publicly available (see the files in Supplementary Materials).

### 3.1. Data Collection

Data collection was made through an Android app developed by the authors that allowed an easy recording, labeling and storage of the data. To do this, we organized an initial data collection that lasted about a month, to see what data we were getting and to be able to do some initial tests on it. Later, we carried out another more intensive collection, over a period of about a week, to alleviate the imbalances and weaknesses found in the previous gathering. Each of the people who took part in the study was asked to set the activity they were going to perform at each moment, through that Android app, before starting the data collection. In this way, once the activity was selected, the gathering of such data was automatically started, until the user indicated the end of such activity. Hence, each stored session corresponds to a specific activity, carried out by a particular individual. Regarding the activities performed, they were four:

- Inactive: not carrying the mobile phone. For example, the device is on the desk while the individual performs another kind of activities.
- Active: carrying the mobile phone, moving, but not going to a particular place. In other words, this means that, for example, making dinner, being in a concert, buying groceries or doing the dishes count as “active” activities.
- Walking: Moving to a specific place. In this case, running or jogging count as a “walking” activity.
- Driving: Moving in a means of transport powered by an engine. This would include cars, buses, motorbikes, trucks and any similar.

The data collected comes from four different sensors: accelerometer, gyroscope, magnetometer and GPS. We selected accelerometer and gyroscope because they are the most used in the literature and the ones that showed the best results. We also added the magnetometer and GPS because we think they could be useful in this problem. In fact, in our case, GPS should be essential to differentiate the activities performed by being able to detect the user’s movement speed who carries the smartphone.

We save the data of the accelerometer, the gyroscope and the magnetometer with their tri-axial values. In the case of GPS, we store the device’s increments in latitude, longitude and altitude, as well as the bearing, speed and accuracy of the collected measurements. Also, for the accelerometer, we used the gravity sensor, subtracting the last reading of the latter from the observations of the first. In this way, we get clear accelerometer values (linear accelerometer), as they are not affected by the smartphone’s orientation. Therefore, we obtain a dataset independent of the place where the individual is, as well as of the device’s bearings.

On the other hand, before saving the data locally, a series of filters are applied. In the case of the accelerometer and magnetometer, we use a low-pass filter to avoid too much noise in these sensor’s measurements. Concerning the gyroscope, to bypass the well-known gyro drift, a high-pass filter was used instead. Nevertheless, we also had to deal with Android’s sensor frequency problem, as we cannot set the same frequency for each one of them. In our case, this is especially problematic, having to join data from very high-frequency sensors such as the accelerometer, with a low-frequency sensor, such as the GPS. From the latter, we obtain new measurements every ten seconds, approximately, compared to the possible ten, or even 50, measurements per second we can get from the accelerometer. Anyhow, given the inability to set a frequency in Android and having to take the values as they are offered by the system itself, there may be gaps in the measurements. These gaps are especially problematic in the case of GPS, where there may be cases where no new measurements were obtained in more than a minute (although perhaps this is mainly due to the difficulty of accessing closed environments). Such gaps also occur in the case of the accelerometer, gyroscope or magnetometer, despite offering about 10, 5 or 8 measurements per second, respectively, in the most stable cases. In these cases, the gaps are between 1 and 5 s, and occur mostly at the start of each data collection session, although much less frequently than with GPS. In this way, in Table 2, we show the average number of recordings per second for each sensor and each activity measured, as well as the resulting average frequency. Below each average value, in a smaller size, we also show the standard deviation for each class. Please note that for moving activities such as “active” or “walking” there is an increase in these measurements, especially with the accelerometer. This is because the smartphone detects these movements and, to get the most information, its frequency is increased automatically to get the maximum number of measurements. However, this increase also occurs during “driving” activity, even more so. Vibrations due to the car use may be the cause of this increase, as they might also be detected by the sensors of the smartphone. Additionally, in “walking” and “active” activities there may be certain inactive intervals (like waiting for a traffic light to go green or just standing doing something, respectively) that lower these averages.

In this way, the final distribution of the activities in our dataset is the one shown in Table 3. In this table, we measured the total time recorded, the number of recordings, the number of samples and the percentage of data (this one related to the number of samples), for each of the activities we specified. Here, each recording refers to a whole activity session, since the individuals begin an action until they stop it; while each sample is related to a single sensor measurement. As can be seen, there are less samples on “inactive” activities in proportion to the total time recorded. This is because the frequency of the sensors increases with activities that require more movement, as explained above, so in these cases they remained at a lower value. Therefore, the total percentage of the data may give a wrong view of the total data distribution, once the sliding windows are applied. This is because, by using these windows on which to compute a series of features, the number of samples actually moves into second place, with the total time recorded being the most important value. The more total time recorded, the more sliding windows computed, and the more patterns for that class. Hence, there would be a much clearer imbalance in the dataset, where “inactive” activity would have three times as many patterns as in the case of “walking”. Regarding the number of recordings made, there are far more with the “walking” activity than with the rest. Anyhow, we consider that the dataset remains useful and feasible to implement models that could distinguish these activities. Moreover, the total number of individuals who participated in the study was 19. Therefore, the dataset also contains different kinds of behaviors that end up enriching the possible models developed later.

On the other hand, there is also another problem in Android, as not all devices contain a gyroscope or a magnetometer to this day. While it is mandatory to have an accelerometer and a GPS, a gyroscope or a magnetometer are not compulsory in older versions of Android. In this way, some of our users took measurements without including these sensors. In Table 4 and Table 5, we show the number of samples that do not include a gyroscope or a gyroscope and a magnetometer simultaneously, as the people who did not have a magnetometer did not have a gyroscope either. Something important to highlight in these tables is the difference in the relation between the number of samples and the time recorded compared to the one showed in Table 3. Here, the number of samples is much higher in relation to the time recorded. This may explain the strange data that we pointed out before in Table 2, as the accelerometer may increase more its frequency in general, by becoming the only sensor to detect motion. On another note, the percentages we show in this table are related to the whole amount of data, from Table 3. Fortunately, these percentages are quite low, and the dataset is not as affected by this problem. Anyhow, it will be something to keep in mind when preparing the data to be applied to a future AI model.

### 3.2. Data Preparation and Feature Extraction

After having collected the data, we proceed to prepare them to be introduced later in the model. To do so, and taking into account the well-known time-series segmentation problem in HAR, we opted to use sliding windows of 20 s, with an overlap of 19 s (95%). We chose 20 s because it is the most we have seen used in this field. Moreover, we consider that our activities, being long-themed, need a large window size to be correctly detected. We thought even a greater size could be beneficial, but we decided to be conservative and see what happens with a smaller one. As for the overlap, we chose the maximum possible that would allow us to have comfortable handling of the data, as well as a higher number of patterns, with one second between windows. In this way, we get around half a million patterns, on a quite long time window, compared to previous works. Additionally, with this distribution, we hope to get reliable results for the movements we are analyzing, as they are long-themed (inactive, active, walking and driving).

However, to apply these windows, it is first necessary to pre-process the data. The algorithm implemented to do so consists of deleting rows that met one or more of the following properties:

1. GPS increments in latitude, longitude and altitude that are higher than a given threshold, obtained from a prior, and very conservative, data study. We detected that there were occasional “jumps” in our GPS-related values, as some of these observations were outside the expected trajectory. For this reason, we decided to fix a threshold of 0.2 for latitude and longitude increments, and 500 for the altitude ones. In this way, any value that is too far out of line is eliminated, keeping those that are closer to the expected.
2. Timestamps that do not match the structure defined (*yyyy-MM-dd HH:mm:ss.ZZZ*) or that do not correspond to an actual date (year 1970 values, for example).
3. Any misplaced value between timestamp and *z*-axis magnetometer, which showed to appear in some very few observations at the beginning of the project.

Table 6 shows the mean and standard deviation values of each sensor for each of the activities studied, after the application of this algorithm. To correctly understand the values indicated in this table, it is important to explain what each of these sensors measures. The accelerometer values correspond to the acceleration force applied to the smartphone on the three physical axes (x,y,z), in m/s${}^{2}$. On the other hand, the gyroscope measures in rad/s the smartphone’s rotation speed around each of the three physical axes (x,y,z). Regarding the magnetometer, it measures the environmental geomagnetic field of the three physical axes (x,y,z) of the smartphone, in μT. As for the GPS, its values correspond, on the one hand, to the increments of the values of the geographical coordinates, longitude and latitude, in which the smartphone is located, with respect to the previous measurement. Similarly, the increments in altitude, in meters, were also measured. Then, the values of speed, bearing and accuracy were also taken into account. Speed was measured in m/s and specifies the speed that is taking the smartphone. The bearing measured the horizontal direction of travel of the smartphone, in degrees. Finally, accuracy values refer to the deviation from the actual smartphone location, in meters, where the smaller the value, the better the accuracy of the measurement. Going back to Table 6, in each cell, the values corresponding to the mean are at the top and, at the bottom, in a smaller size, the standard deviation values. Each pair of values corresponds to the set that forms each sensor. In the case of the accelerometer, gyroscope and magnetometer, these refer to the values related to their “*X*”, “*Y*” and “*Z*” axes. As for the GPS, this set is formed by the latitude increments (Lat.), the longitude increments (Long.), the altitude increments (Alt.), the speed (Sp.), the bearing (Bear.) and the accuracy (Acc.) of every measurement. Here, it is worth noting some rare data, such as those relating to GPS “inactive” activity, where the values are very high concerning what is expected from such action. In this case, we consider that these values are due to the fact that such activity is carried out in indoor environments, which are not so accessible for GPS. Even so, as can be seen, there are some clear differences between the activities, so the possibilities of identification with future models are more than feasible.

After applying previous preprocessing, since data collection required the user to tap a button before performing the activity, we eliminated the first five seconds of each activity collection. In the same way, we did so with the final five seconds of each measurement. Hence, we can prevent the future models from ending up learning the movement that precedes the start or the end of the action, such as, for example, putting the smartphone in the pocket or pulling it out. While doing this, we also take each recorded activity and split it into the previously defined time interval to prepare them for the next step. The remaining parts of each period are discarded. In this way, in Table 7, the final results after the application of this sliding window and overlap is shown for the samples containing all the sensors. As we already addressed in the previous section, although at the sample level the data may appear lower for activities such as inactive or walking, at the final pattern level the results are much different.

Later, we had to go through a transformation process to extract the features and apply all the information needed for the classification algorithm. Due to GPS’ low frequency, to carry out this feature extraction, it was necessary to previously replicate some of the data stored by this sensor, for each of the windows applied. To do this, if the difference between one observation and the next differed in a longer time than one second, the latter measurement is replicated, with a different timestamp. For this reason, all sessions that do not contain at least one GPS observation are removed from the list of valid ones for this process. We repeat this step until all the windows that may be in the middle are correctly filled. We selected one second as the amount of time to be between each sample, so there is always at least one observation in each of the windows applied, making the final feature extraction match to the data obtained. After that, for each set of measurements, we computed six different types of features, each generating a series of inputs for the AI model. The features used were: mean, variance, median absolute deviation, maximum, minimum and interquartile range, all based in the time domain. All of them were used in previous works like [16], with remarkable results. In this way, we maintain the simplicity of the model, being able to complicate it or change it in future works according to the results we achieve.

### 3.3. Classification Algorithm

As already indicated in the related work section, there are many kinds of models used in HAR. In our case, we chose to employ an SVM model. Although SVM showed excellent results with rather short-themed activities, we consider it interesting to test it as an initial model in our dataset. It is one of the most used models in HAR, applied in works such as [9,16] and, more recently, in [23], all with outstanding overall performance in this field, as well as being a simple and straightforward AI model.

An SVM is a supervised machine learning model that uses classification algorithms for two-group classification problems. After giving an SVM model tagged training data sets for either category, they can categorize new examples. To do this, the SVM looks for the hyperplane that maximizes the margins between the two classes. In other words, it looks for the hyperplane whose distance from the nearest element in each category is the highest. Hither, non-linearity is achieved through kernel functions, which implicitly map the data to a more dimensional space where this linear approximation is applied. On the other hand, other hyperparameters such as C or gamma also affect the definition of this hyperplane. As for C, it marks the width of the margins of this hyperplane, as well as the number of errors that are accepted. Concerning gamma, it directly affects the curve of the hyperplane, making it softer or more accentuated, depending on the patterns that are introduced into the model.

While SVM is typically used to solve binary classification tasks, it can also be used in multi-class problems. To do this, it is necessary to use a *one-vs-all* or *one-vs-one* strategy. The first case is designed to model each class against all other classes independently. In this way, a classifier is created for each situation. On the other hand, the second case is used to model each pair of classes separately, performing various binary classifications, until a final result is found. In our case, we will be using a one-vs-all approach, as it is the most used one in the literature. For this, we implemented it on Python, using the functions provided with Scikit-learn.

## 4. Results and Discussion

### 4.1. Results

To provide reliable results in this dataset to future users, we conducted a series of experiments on it. For this purpose, we applied SVM classifiers, looking for the best kernel between Polynomial, RBF (Radial Basis Function) and Linear SVM. Also, we explored the optimal trade-off parameter C, the bandwidth γ in RBF and Polynomial kernels, as well as the degree in this last one, with the features discussed in the previous section. The reason we selected these kernels was, on the one hand, because the RBF kernel is one of the most used ones in the literature. On the other hand, the linear and the polynomial ones were also selected to have a basis for comparison. To select the best configuration and architecture of the network, we obeyed the following organization:

1. First, with the whole combination of all sensors, we made a stratified 10-fold with which to have 10 sets with presumably the same number of patterns for each class.
2. Then, we took each of those folds to use them to perform a grid search on their corresponding dataset. To evaluate the resulting predictions, since we use a one-vs-all approach that will have unbalanced data in each sub-classifier, we chose the f1-score metric to minimize this problematic. The f1-score is a measure of the test accuracy, based on the harmonic mean of the precision and the recall metrics. Its formula would be as follows:
  $$F_{1}=2\times \frac{\mathrm{Precision}\times \mathrm{Recall}}{\mathrm{Precision}+\mathrm{Recall}}$$
  With that in mind, it is closely linked to the correct classification of each pattern, not being so influenced by class imbalances. When this happens, accuracy might give an incorrect idea of the model’s performance. However, the f1-score will give a slightly smoother value that better represents that model, making it a good option for our grid search. On the other hand, we also set a maximum number of iterations (1000) as a stop criterion, given the high-dimensional data and the scaling problem of SVM. To carry out this process, we selected the following hyperparameters: as kernels, we chose the polynomial, the RBF and the linear ones, because of what we addressed before. As for parameter C, we selected those of the set {1, 10, 100, 1000, 10000}. For the γ parameter, specifically for the RBF and polynomial kernels, we chose those of the set {0.0001, 0.001, 0.01, 0.1, 1}. Concerning the degree parameter for the polynomial kernel, we selected those of the set {1, 2, 3, 4}.
3. Once the grid search is done, we evaluated the results and selected the best combination of hyperparameters for each fold. Then, we tested the best corresponding model.
4. Finally, we studied the impact of the gyroscope and magnetometer, taking advantage of the users that could not include these sensors in their measurements. For this purpose, we prepared three different sets: accelerometer + gyroscope + magnetometer + GPS (all users but the ones missing gyroscope and magnetometer), accelerometer + gyroscope + GPS (all users but the ones missing magnetometer) and accelerometer + GPS (all users).

The first steps of the experiments yielded the results that can be seen in Table 8. In this table, for each cell, we show the average test f1-score obtained (top), as well as its standard deviation (below). As can be seen, the best results correspond, in general, to the RBF kernel, and, more specifically, for cases where γ equals 0.01, especially in conjunction with C = 10. With this combination of hyperparameters, we managed to achieve an f1-score of 64.14%.

The average confusion matrix yielded by the third step of the experiments is the one showed in Table 9, along with its particular metrics (recall, precision and accuracy). This result corresponds to an accuracy of 67.22%. As can be seen, the model manages to correctly separate “inactive” events but struggles with the rest, especially with the “active” one. In this case, we think that this is due to the diffusion of this action since it combines both moments of inactivity and movement, in which we may walk from one place to another. On the other hand, we can also see that the activities of “walking” and “driving” are also confused with each other. This was expected considering that most driving took place in an urban environment. In this scenario, there may be traffic jams or moments of less fluidity that may be quite similar, at a sensory level, to the data obtained while performing the “walking” activity, as well as the rest of actions. Anyhow, the GPS is probably very influential in this confusion and it would be interesting to change the related features used to see how they affect the final classification. Maybe greater sliding window sizes or any kind of feature related to the Fourier transform of the signal, to pick up its periodic component, could positively affect the final model.

To a lesser extent, it is also important to note that there are some cases in which some activities are confused as an “inactive” action. This was also relatively expected, as every activity is subject to prolonged stoppages. For example, while acting as “walking” or “driving”, traffic lights that force the individual to stop may appear. In these situations, these pauses may be mistaken by the model for cases of pure inactivity. Perhaps the use of other and more specific features could improve the differentiation in all these cases, as well as the use of another type of AI algorithms and bigger sliding window sizes.

Regarding the fourth and last step, we also applied the same algorithm for the rest of the data sets formed, obtaining the results shown in Table 10. Similar to the other tables shown, the average values are on the left side of each cell, while the standard deviations are on the right side, in a smaller size. This comparison is made from the average of the test values yielded by the experiments conducted to each set. As can be seen, the combination of the four sensors performs better in comparison with the other two, especially with the case formed only by accelerometer and GPS. Both the gyroscope and the magnetometer seem to have a pretty important implication for the final classification. In the first case, it seems to significantly improve the final accuracy, as in the other works that included it in their studies. However, it looks like what makes the highest difference is the appendage of this sensor to the magnetometer.

### 4.2. Discussion

Although the results obtained might not seem as good as those seen so far in the rest of the literature, we consider that they are promising given the problem addressed. The data used are very different from those of the other datasets that currently exist in the field, as well as being much less specific. Therefore, while the results may seem worse, actually they are not comparable. The data collected correspond to different profiles of people, each with their physical peculiarities and ways of using their smartphone. Moreover, the nature of each of the defined activities implies short periods of some of the other actions. For example, within the “active” exercise, there are both moments of inactivity and moments of travel. Within the “walking” activity, there may be stops due to traffic lights or other obstacles encountered along the way. Furthermore, during the action of “driving”, it is noteworthy that an urban environment has many peculiarities and stops that can complicate the final classification. Therefore, given these problems and the simplicity of the proposed model, we consider that these results are a relatively good first approximation of what they could be. We believe that perhaps with other types of models also used in this field, such as Random Forest, the results could be improved considerably. Also, through the application of algorithms based on deep learning, such as LSTM, that showed exceptional performance in this domain too. Hence, with this change in the model to be used and the addition of new metrics, we would surely get closer to that real-life environment we are searching.

## 5. Conclusions and Future Work

In this paper, we presented a dataset for the HAR field. This dataset contains information from 19 different users, each with its own way of using their smartphone, as well as their physical peculiarities. The amount of data is enough to make classifications about them, and the information gathered is realistic enough to be taken to a real-life environment.

Therefore, with the development of this dataset, we hope to alleviate the problems that are seen in other works. While it is true that the final results we got may not be as good as those seen to date, we believe that it will be the beginning of the road to take the models developed for HAR to real life. We also hope that the current confusions of the proposed model, among some of the determined activities, can be overcome in future research. In this way, it would be possible to implement a system capable of correctly detecting a person’s movements or activities, regardless of the way they use their smartphone or their physical peculiarities. This could be very interesting for many companies or individuals to be able to monitor or predict the activities performed by a particular individual.

For this reason, we will continue advancing in the same line of work, testing other techniques that also had pretty good results in the field, such as Random Forest, CNN or LSTM. Also, the deletion or the addition of new features, such as those related to the Fourier transform, to search for possible periodic components in the stored signals, could positively affect the final model. In this way, we will be able to compare the results obtained, in search of the best model to solve this problem. In addition, we will also explore the real impact of the sensors used, as well as other possible sliding windows greater sizes and combinations of hyperparameters, in search of improving the best configuration found so far.

## Figures and Tables

**Table 1:** Comparison between datasets: UCI HAR, WISDM and the proposed one.

	UCI HAR	WISDM	Proposed
**Type of actions studied**	Short-themed	Short-themed	**Long-themed**
**Smartphone orientation and positioning**	Fixed	Fixed	**Free**
**Different individuals**	Yes	Yes	**Yes**
**Fixed sensor frequency**	Yes	Yes	**No**
**Sensors used**	Acc. and gyro.	Acc. and gyro.	**Acc., gyro., magn. and GPS**

**Table 2:** Average number of recordings per second for each sensor and each activity measured.

Activity	Accelerometer Hz.	Gyroscope Hz.	Magnetometer Hz.	GPS Hz.
**Inactive**	11.00 ±16.38	4.66 ±0.74	7.91 ±11.72	0.13 ±0.35
**Active**	32.55 ±24.80	4.46 ±1.44	9.13 ±13.64	0.06 ±0.23
**Walking**	31.24 ±27.47	6.24 ±11.86	8.16 ±12.05	0.06 ±0.23
**Driving**	51.16 ±31.59	4.66 ±2.42	17.00 ±20.01	0.04 ±0.20

**Table 3:** Dataset distribution for each activity measured.

Activity	Time Recorded (s)	Number of Recordings	Number of Samples	Percentage of Data
**Inactive**	292,213	147	7,064,757	24.25%
**Active**	178,806	99	8,918,021	30.62%
**Walking**	98,071	200	4,541,130	15.59%
**Driving**	112,226	128	8,602,902	29.54%
**Overall**	681,316	574	29,126,810	100%

**Table 4:** Dataset distribution for each activity measured without gyroscope.

Activity	Time Recorded (s)	Number of Recordings	Number of Samples	Percentage of Data
**Inactive**	11,523	8	668,536	2.29%
**Active**	13,866	7	619,913	2.13%
**Walking**	4169	15	584,262	2.01%
**Driving**	25,718	23	3,776,468	12.97%
**Overall**	55,276	53	5,649,179	19.40%

**Table 5:** Dataset distribution for each activity measured without gyroscope and magnetometer.

Activity	Time Recorded (s)	Number of Recordings	Number of Samples	Percentage of Data
**Inactive**	5409	2	269,710	0.93%
**Active**	10,286	2	90,487	0.31%
**Walking**	0	0	0	0%
**Driving**	0	0	0	0%
**Overall**	25,695	4	360,197	1.24%

**Table 6:** Sensor’s mean and standard deviation values for each activity measured.

		Activity			
		Inactive	Active	Walking	Driving
	*X*	0.11761 ±0.45934	−0.01338 ±1.30277	0.09425 ±3.33422	−0.04747 ±0.83290
**Accelerometer**	*Y*	0.06136 ±0.26764	0.07598 ±1.45440	−0.37604 ±4.35808	−0.12936 ±0.93828
	*Z*	0.84318 ±2.66926	0.13008 ±1.70294	0.07353 ±4.09859	0.18127 ±1.24042
	*X*	−0.00004 ±0.03828	−0.00001 ±0.36806	0.00760 ±1.31125	0.00080 ±0.19224
**Gyroscope**	*Y*	0.00004 ±0.04719	−0.00102 ±0.40959	−0.00020 ±0.89244	0.00277 ±0.19835
	*Z*	0.00001 ±0.03526	0.00055 ±0.24528	−0.00560 ±0.53685	−0.00243 ±0.16678
	*X*	25.93805 ±56.45617	6.03153 ±30.00980	−0.28182 ±27.03210	−5.96356 ±46.08005
**Magnetometer**	*Y*	−19.62683 ±85.70343	−0.02890 ±28.76398	18.73800 ±29.63926	10.73609 ±40.46829
	*Z*	−56.60425 ±33.19593	9.56310 ±39.76136	0.64541 ±25.55331	−2.93043 ±29.45994
	**Lat.**	0.00075 ±0.00166	0.00112 ±0.00234	0.00047 ±0.00220	0.00175 ±0.00365
	**Long.**	0.00125 ±0.00285	0.00118 ±0.00314	0.00056 ±0.00300	0.00204 ±0.00420
**GPS**	**Alt.**	32.59169 ±53.06269	30.77538 ±48.65634	34.06931 ±42.51933	41.59391 ±54.74934
	**Sp.**	0.37222 ±0.82495	0.12109 ±0.81007	0.79924 ±0.71835	10.82191 ±11.82733
	**Bear.**	57.25005 ±105.49576	14.69719 ±56.00693	124.85103 ±119.80663	118.88108 ±118.78510
	**Acc.**	265.44485 ±494.66499	214.57640 ±429.81169	75.54539 ±259.59907	192.90736 ±508.87285

**Table 7:** Number of patterns for the samples containing all the sensors with a sliding window of 20 s and 19 s overlap.

Activity				
**Inactive**	**Active**	**Walking**	**Driving**	**Overall**
201,501	137,407	86,383	77,852	503,143
(40%)	(27%)	(17%)	(16%)	

**Table 8:** Mean f1-scores achieved for each combination of kernel, C, γ and degree hyperparameters in the grid search. The best result found is highlighted in bold.

		C = 1	C = 10	C = 100	C = 1000	C = 10,000
**Linear**		36.15% ±15.45	31.41% ±12.78	31.41% ±12.78	31.41% ±12.78	31.41% ±12.78
	γ = **0.0001**	10.56% ±13.25	4.57% ±0.42	17.04% ±9.20	40.72% ±16.80	34.70% ±13.68
	γ = **0.001**	20.67% ±14.81	21.30% ±19.99	39.71% ±16.41	38.70% ±20.79	46.70% ±17.60
**RBF**	γ = **0.01**	60.37% ±12.76	**64.14%** ±19.66	56.47% ±15.95	57.20% ±16.79	56.49% ±14.14
	γ = **0.1**	51.76% ±12.00	54.10% ±14.91	57.09% ±13.24	51.62% ±14.97	51.36% ±15.18
	γ = **1**	±12.84	41.16% ±12.58	41.28% ±12.65	41.28% ±12.65	41.28% ±12.65
	γ = **0.0001**	18.09% ±13.92	21.04% ±18.97	41.00% ±19.70	32.67% ±10.93	37.12% ±16.61
	γ = **0.001**	16.09% ±8.09	37.86% ±16.86	37.82% ±14.72	37.26% ±18.32	32.01% ±13.80
**Poly d=1**	γ = **0.01**	37.73% ±18.58	41.49% ±17.97	36.16% ±12.30	36.67% ±12.98	36.67% ±12.98
	γ = **0.1**	33.36% ±15.56	32.58% ±13.87	34.11% ±12.32	34.11% ±12.32	34.11% ±12.32
	γ = **1**	36.15% ±15.45	31.41% ±12.78	31.41% ±12.78	31.41% ±12.78	31.41% ±12.78
	γ = **0.0001**	10.96% ±2.27	6.27% ±2.76	7.03% ±5.52	9.34% ±8.00	9.60% ±9.07
	γ = **0.001**	7.03% ±5.52	9.10% ±7.52	8.39% ±6.12	10.62% ±4.09	22.55% ±6.75
**Poly d=2**	γ = **0.01**	9.60% ±9.07	10.55% ±3.65	23.08% ±7.16	24.34% ±6.93	27.69% ±7.74
	γ = **0.1**	22.73% ±6.26	23.46% ±4.99	25.84% ±6.67	25.82% ±6.64	25.82% ±6.64
	γ = **1**	25.58% ±8.47	25.59% ±8.46	25.59% ±8.46	25.59% ±8.46	25.59% ±8.46
	γ = **0.0001**	6.11% ±2.83	6.86% ±3.19	10.61% ±6.90	9.15% ±5.78	11.16% ±5.29
	γ = **0.001**	9.15% ±5.78	11.16% ±5.29	6.04% ±3.64	8.56% ±4.89	19.86% ±9.13
**Poly d=3**	γ = **0.01**	8.32% ±5.16	23.63% ±7.98	23.18% ±9.30	20.63% ±6.19	30.29% ±18.15
	γ = **0.1**	21.79% ±8.69	25.40% ±15.24	27.70% ±14.57	27.70% ±14.57	27.70% ±14.57
	γ = **1**	23.11% ±15.45	23.11% ±15.45	23.11% ±15.45	23.11% ±15.45	23.11% ±15.45
	γ = **0.0001**	7.33% ±5.60	8.20% ±3.53	6.96% ±3.13	4.78% ±0.41	10.36% ±7.03
	γ = **0.001**	10.36% ±7.03	7.63% ±5.89	7.84% ±5.79	13.20% ±8.45	9.68% ±8.53
**Poly d=4**	γ = **0.01**	9.68% ±8.61	9.54% ±8.82	8.04% ±5.00	7.11% ±3.37	11.79% ±8.47
	γ = **0.1**	8.39% ±3.41	12.34% ±8.48	12.34% ±8.48	12.34% ±8.48	12.34% ±8.48
	γ = **1**	9.02% ±5.63	9.02% ±5.63	9.02% ±5.63	9.02% ±5.63	9.02% ±5.63

**Table 9:** Average confusion matrix for the experiments conducted.

		Ground Truth					
	Inactive	Active	Walking	Driving	Precision
Inactive	15,887	1904	1165	1195	78.84%
Active	3226	6159	3134	1222	44.82%
Walking	259	1540	5863	976	67.88%
Driving	149	653	1073	5910	75.92%
Recall	81.38%	60.05%	52.19%	63.53%	67.22%

**Table 10:** Mean accuracies achieved for each set of data, with the best group result highlighted in bold.

Acc. + GPS.	Acc. + Magn. + GPS	Acc. + Gyro. + Magn.+ GPS
60.10% ±11.43	62.66% ±11.68	**67.22%** ±13.13

## Supplementary Materials

The complete dataset, as well as the scripts used on our experiments, are available online at http://lbd.udc.es/research/real-life-HAR-dataset. Similarly, they have also been uploaded to Mendeley Data [24].
