# Correction: Garcia-Gonzalez, D.; Rivero, D.; Fernandez-Blanco, E.; Luaces, M.R. A Public Domain Dataset for Real-Life Human Activity Recognition Using Smartphone Sensors. *Sensors* 2020, *20*, 2200

**DOI:** 10.3390/s20164650

**Published:** 2020-08-18

**Authors:** Daniel Garcia-Gonzalez, Daniel Rivero, Enrique Fernandez-Blanco, Miguel R. Luaces

**Affiliations:** Department of Computer Science and Information Technologies, University of A Coruna, 15071 A Coruna, Spain; daniel.rivero@udc.es (D.R.); enrique.fernandez@udc.es (E.F.-B.); miguel.luaces@udc.es (M.R.L.)

The authors wish to make the following corrections to this paper [1]:

## 1. Changes in Main Body Paragraphs

The authors are sorry to report that an error was found in the processing of data, before the SVM application. What was happening was that the 20-second windows they were commenting on were not implemented correctly, as they were not always of that length. Once they found and solved the error, they executed everything again to get those preliminary results that were proposed in the paper. For that reason, the values obtained since that moment change. In addition, there were some activity sessions that had some time gaps that made them defective, which were not detected before. Hence, these sessions were ignored. Consequently, the authors wish to make at this time the following corrections to the paper:

- On page 8, at the end of the “Data Preparation and Feature Extraction” section, the sentences “While doing this, we also take each recorded activity and split it into the previously defined time interval to prepare them for the next step. The remaining parts of each period are discarded.” should be “While doing this, we also get rid of those sessions that have quite large gaps between the data (at least five seconds) for any sensor other than the GPS, by considering them as invalid.”
- Following the previous sentences, there is a typo on the next one, where “In this way, in Table 7, the final results after the application of this sliding window and overlap is shown for the samples containing all the sensors.” should be “In this way, in Table 7, the final results after the application of this sliding window and overlap are shown for the samples containing all the sensors.”
- On page 10, in the “Results” section, the sentences “As can be seen, the best results correspond, in general, to the RBF kernel, and, more specifically, for cases where *γ* equals 0.01, especially in conjunction with C = 10. With this combination of hyperparameters, we managed to achieve an f1-score of 64.14%.” should be “As can be seen, the best results correspond, in general, to the RBF kernel, and, more specifically, for cases where *γ* equals 0.1, especially in conjunction with C = 100. With this combination of hyperparameters, we managed to achieve an f1-score of 74.34%.”
- Following the previous sentences, in the next paragraph, the sentence “This result corresponds to an accuracy of 67.22%.” should be “This result corresponds to an accuracy of 69.28%.”
- On page 12, at the end of the “Results” section, the sentences “As can be seen, the combination of the four sensors performs better in comparison with the other two, especially with the case formed only by accelerometer and GPS. Both the gyroscope and the magnetometer seem to have a pretty important implication for the final classification. In the first case, it seems to significantly improve the final accuracy, as in the other works that included it in their studies. However, it looks like what makes the highest difference is the appendage of this sensor to the magnetometer.” should be “As can be seen, the combination of the accelerometer, the magnetometer and the GPS, with the lack of the gyroscope, performs better in comparison with the other two, especially with the case formed only by accelerometer and GPS. However, the expected best result would have been the one that appends the gyroscope too, as in the other works that included it in their studies. Perhaps the fact that we are studying long-themed activities is something in which the gyroscope does not have much of a presence. In addition, the model has more patterns with the winning combination, which could also positively influence the final result.”

## 2. Changes in Tables

Regarding the error commented above, with new data windows, the outcomes of the model are different. For that reason, every value shown in the tables since the processing of data differs. Therefore, replace:

**Table 7:** Number of patterns for the samples containing all the sensors with a sliding window of 20 s and 19 s overlap.

Activity				
**Inactive**	**Active**	**Walking**	**Driving**	**Overall**
201,501	137,407	86,383	77,852	503,143
(40%)	(27%)	(17%)	(16%)	

with:

**Table 7:** Number of patterns for the samples containing all the sensors with a sliding window of 20 s and 19 s overlap.

Activity				
**Inactive**	**Active**	**Walking**	**Driving**	**Overall**
214,130	140,060	83,376	61,710	499,276
(43%)	(28%)	(17%)	(12%)	

For the same reason, replace:

**Table 8:** Mean f1-scores achieved for each combination of kernel, C, *γ* and degree hyperparameters in the grid search. The best result found is highlighted in bold.

		C = 1	C = 10	C = 100	C = 1000	C = 10,000
**Linear**		36.15% ±15.45	31.41% ±12.78	31.41% ±12.78	31.41% ±12.78	31.41% ±12.78
	**γ = 0.0001**	10.56% ±13.25	4.57% ±0.42	17.04% ±9.20	40.72% ±16.80	34.70% ±13.68
	**γ = 0.001**	20.67% ±14.81	21.30% ±19.99	39.71% ±16.41	38.70% ±20.79	46.70% ±17.60
**RBF**	**γ = 0.01**	60.37% ±12.76	**64.14%** **±19.66**	56.47% ±15.95	57.20% ±16.79	56.49% ±14.14
	**γ = 0.1**	51.76% ±12.00	54.10% ±14.91	57.09% ±13.24	51.62% ±14.97	51.36% ±15.18
	**γ = 1**	50.99% ±12.84	41.16% ±12.58	41.28% ±12.65	41.28% ±12.65	41.28% ±12.65
	**γ = 0.0001**	18.09% ±13.92	21.04% ±18.97	41.00% ±19.70	32.67% ±10.93	37.12% ±16.61
	**γ = 0.001**	16.09% ±8.09	37.86% ±16.86	37.82% ±14.72	37.26% ±18.32	32.01% ±13.80
**Poly d = 1**	**γ = 0.01**	37.73% ±18.58	41.49% ±17.97	36.16% ±12.30	36.67% ±12.98	36.67% ±12.98
	**γ = 0.1**	33.36% ±15.56	32.58% ±13.87	34.11% ±12.32	34.11% ±12.32	34.11% ±12.32
	**γ = 1**	36.15% ±15.45	31.41% ±12.78	31.41% ±12.78	31.41% ±12.78	31.41% ±12.78
	**γ = 0.0001**	10.96% ±2.27	6.27% ±2.76	7.03% ±5.52	9.34% ±8.00	9.60% ±9.07
	**γ = 0.001**	7.03% ±5.52	9.10% ±7.52	8.39% ±6.12	10.62% ±4.09	22.55% ±6.75
**Poly d = 2**	**γ = 0.01**	9.60% ±9.07	10.55% ±3.65	23.08% ±7.16	24.34% ±6.93	27.69% ±7.74
	**γ = 0.1**	22.73% ±6.26	23.46% ±4.99	25.84% ±6.67	25.82% ±6.64	25.82% ±6.64
	**γ = 1**	25.58% ±8.47	25.59% ±8.46	25.59% ±8.46	25.59% ±8.46	25.59% ±8.46
	**γ = 0.0001**	6.11% ±2.83	6.86% ±3.19	10.61% ±6.90	9.15% ±5.78	11.16% ±5.29
	**γ = 0.001**	9.15% ±5.78	11.16% ±5.29	6.04% ±3.64	8.56% ±4.89	19.86% ±9.13
**Poly d = 3**	**γ = 0.01**	8.32% ±5.16	23.63% ±7.98	23.18% ±9.30	20.63% ±6.19	30.29% ±18.15
	**γ = 0.1**	21.79% ±8.69	25.40% ±15.24	27.70% ±14.57	27.70% ±14.57	27.70% ±14.57
	**γ = 1**	23.11% ±15.45	23.11% ±15.45	23.11% ±15.45	23.11% ±15.45	23.11% ±15.45
	**γ = 0.0001**	7.33% ±5.60	8.20% ±3.53	6.96% ±3.13	4.78% ±0.41	10.36% ±7.03
	**γ = 0.001**	10.36% ±7.03	7.63% ±5.89	7.84% ±5.79	13.20% ±8.45	9.68% ±8.53
**Poly d = 4**	**γ = 0.01**	9.68% ±8.61	9.54% ±8.82	8.04% ±5.00	7.11% ±3.37	11.79% ±8.47
	**γ = 0.1**	8.39% ±3.41	12.34% ±8.48	12.34% ±8.48	12.34% ±8.48	12.34% ±8.48
	**γ = 1**	9.02% ±5.63	9.02% ±5.63	9.02% ±5.63	9.02% ±5.63	9.02% ±5.63

with:

**Table 8:** Mean f1-scores achieved for each combination of kernel, C, γ and degree hyperparameters in the grid search. The best result found is highlighted in bold.

		C = 1	C = 10	C = 100	C = 1000	C = 10,000
**Linear**		36.33% ±17.03	38.96% ±12.11	42.58% ±12.54	42.58% ±12.54	42.58% ±12.54
	**γ = 0.0001**	5.88% ±4.28	11.08% ±11.80	17.81% ±7.68	39.94% ±18.53	37.38% ±20.70
	**γ = 0.001**	15.78% ±14.89	28.26% ±18.39	45.12% ±15.03	41.09% ±9.19	42.75% ±14.17
**RBF**	**γ = 0.01**	59.16% ±7.07	63.21% ±14.51	58.66% ±11.77	65.44% ±16.52	59.24% ±13.19
	**γ = 0.1**	68.30% ±10.80	73.33% ±6.62	**74.34%** ±8.26	70.94% ±7.92	69.42% ±9.51
	**γ = 1**	63.73% ±10.69	56.88% ±6.21	56.96% ±6.30	56.96% ±6.30	56.96% ±6.30
	**γ = 0.0001**	12.89% ±9.60	29.65% ±17.48	37.20% ±18.34	26.37% ±13.73	43.37% ±19.91
	**γ = 0.001**	29.39% ±16.57	33.50% ±20.58	34.51% ±15.72	39.10% ±17.36	39.17% ±15.85
**Poly d = 1**	**γ = 0.01**	32.08% ±16.92	33.90% ±11.22	40.71% ±19.78	43.18% ±18.73	39.07% ±18.59
	**γ = 0.1**	33.21% ±21.96	33.69% ±16.93	40.93% ±15.31	36.65% ±14.93	36.65% ±14.93
	**γ = 1**	36.33% ±17.03	38.96% ±12.12	42.58% ±12.54	42.58% ±12.54	42.58% ±12.54
	**γ = 0.0001**	7.92% ±4.43	6.85% ±3.62	10.22% ±8.73	5.92% ±5.05	9.69% ±7.02
	**γ = 0.001**	10.22% ±8.73	5.92% ±5.05	9.70% ±7.02	12.34% ±6.12	24.01% ±7.49
**Poly d = 2**	**γ = 0.01**	9.69% ±7.03	12.27% ±5.78	26.54% ±7.70	22.56% ±5.74	20.64% ±6.93
	**γ = 0.1**	23.63% ±7.41	24.40% ±5.83	26.24% ±7.85	26.23% ±7.85	26.23% ±7.85
	**γ = 1**	27.35% ±10.83	27.33% ±10.84	27.33% ±10.84	27.33% ±10.84	27.33% ±10.84
	**γ = 0.0001**	5.61% ±4.41	6.21% ±4.08	7.45% ±4.55	10.01% ±6.79	10.03% ±6.79
	**γ = 0.001**	10.01% ±6.79	10.03% ±6.79	6.60% ±4.46	8.19% ±7.02	20.48% ±12.54
**Poly d = 3**	**γ = 0.01**	5.87% ±3.97	19.68% ±13.58	24.29% ±9.31	22.63% ±8.17	16.92% ±7.38
	**γ = 0.1**	26.40% ±7.11	17.90% ±8.51	17.60% ±12.79	17.60% ±12.79	17.60% ±12.79
	**γ = 1**	17.77% ±8.63	17.77% ±8.63	17.77% ±8.63	17.77% ±8.63	17.77% ±8.63
	**γ = 0.0001**	5.87% ±3.31	6.42% ±3.93	9.09% ±3.98	8.91% ±8.84	13.12% ±8.93
	**γ = 0.001**	13.12% ±8.93	7.92% ±4.44	6.18% ±3.27	11.03% ±10.01	11.26% ±9.90
**Poly d = 4**	**γ = 0.01**	9.16% ±8.76	7.87% ±6.80	6.45% ±3.21	5.52% ±1.81	7.18% ±4.55
	**γ = 0.1**	8.71% ±4.79	9.55% ±6.75	9.49% ±6.89	9.49% ±6.89	9.49% ±6.89
	**γ = 1**	8.97% ±5.29	8.97% ±5.29	8.97% ±5.29	8.97% ±5.29	8.97% ±5.29

For the same reason, replace:

**Table 9:** Average confusion matrix for the experiments conducted.

	Ground Truth				
	Inactive	Active	Walking	Driving	Precision
Inactive	15,887	1904	1165	1195	78.84%
Active	3226	6159	3134	1222	44.82%
Walking	259	1540	5863	976	67.88%
Driving	149	653	1073	5910	75.92%
Recall	81.38%	60.05%	52.19%	63.53%	67.22%

with:

**Table 9:** Average confusion matrix for the experiments conducted.

	Ground Truth				
	Inactive	Active	Walking	Driving	Precision
Inactive	16,787	610	486	90	93.40%
Active	3026	8676	1163	914	62.97%
Walking	1341	3772	5675	1714	45.39%
Driving	259	948	1015	3453	60.85%
Recall	78.40%	61.95%	68.05%	55.96%	69.28%

For the same reason, replace:

**Table 10:** Mean accuracies achieved for each set of data, with the best group result highlighted in bold.

Acc. + GPS.	Acc. + Magn. + GPS	Acc. + Gyro. + Magn. + GPS
60.10% ±11.43	62.66% ±11.68	**67.22% ±13.13**

with:

**Table 10:** Mean accuracies achieved for each set of data, with the best group result highlighted in bold.

Acc. + GPS.	Acc. + Magn. + GPS	Acc. + Gyro. + Magn. + GPS
67.53% ±6.33	**74.39% ±10.75**	69.28% ±15.10

## 3. Changes in References

The authors would like to update the following reference, as it is the Mendeley repository in which both the scripts and data were uploaded. It has been updated with the correction of the error found. Therefore, replace:

- Garcia-Gonzalez D.; Rivero, D.; Fernandez-Blanco, E.; R.Luaces, M. A Public Domain Dataset for Real-Life Human Activity Recognition Using Smartphone Sensors. Mendeley Data 2020, V1, doi:10.17632/3xm88g6m6d.1

with:

- Garcia-Gonzalez, D.; Rivero, D.; Fernandez-Blanco, E.; Luaces, M.R. A Public Domain Dataset for Real-Life Human Activity Recognition Using Smartphone Sensors. Available Online: https://data.mendeley.com/datasets/3xm88g6m6d/2 (accessed on 18 August 2020).

These changes have no material impact on the conclusions of our paper. The authors would like to apologize for any inconvenience caused to the readers by these changes.
